# Top-Down Proteomics in the Assessment of Kidney Donor Quality: A Proof of Concept for Increased Organ Utilization

**DOI:** 10.1016/j.mcpro.2026.101664

**Published:** 2026-09-19

**Authors:** Claudia Ctortecka, Dinesh Jaishankar, Pei Su, Che-Fan Huang, Indira Pla, Nathaniel Henning, Michael A.R. Hollas, Michelle A. Callegari, Meredith E. Taylor, Yu Min Lee, Amna Daud, David F. Pinelli, Vinayak Rohan, Michael A. Caldwell, Eleonora Forte, Aniel Sanchez, Neil L. Kelleher, Satish N. Nadig

**Affiliations:** 1 Chemistry of Life Processes Institute, Northwestern University, Evanston, Illinois, USA; 2 Division of Organ Transplant, Department of Surgery, Feinberg School of Medicine, Northwestern University, Chicago, Illinois, USA; 3 Comprehensive Transplant Center, Feinberg School of Medicine, Northwestern University, Chicago, Illinois, USA; 4 Proteomics Center of Excellence, Northwestern University, Evanston, Illinois, USA; 5 Department of Chemistry, Northwestern University, Evanston, Illinois, USA; 6 Department of Biochemistry, University of California, Riverside, California, USA; 7 Division of Nephrology, Department of Medicine, College of Medicine, University of Illinois Chicago, Chicago, Illinois, USA; 8 Department of Molecular Biosciences, Northwestern University, Evanston, Illinois, USA; 9 Department of Microbiology-Immunology and Pediatrics, Feinberg School of Medicine, Northwestern University, Chicago, Illinois, USA

**Keywords:** top-down proteomics, proteoform imaging, transplantation, kidney biopsy, kidney donation, cell signaling

## Abstract

A persistent barrier to optimizing the utilization of deceased donor kidneys, nearly 30% of which are discarded, is the absence of objective, molecularly resolved metrics of organ quality. To address this gap, we integrated high-resolution proteoform imaging mass spectrometry (PiMS) of donor kidney biopsies with deep LC-MS–based intact proteoform profiling of recipient peripheral blood mononuclear cells (PBMCs) and standard clinical assays. This approach enables direct delineation of proteoform landscapes and reveals immunological and cell stress signatures. Across donor types, living donor (LD) kidneys were enriched for proteoforms associated with oxidative metabolism and cellular homeostasis, whereas kidneys from donation after brain death (DBD), donation after circulatory death (DCD), and discarded organs exhibited elevated abundance of stress-responsive proteoforms, including acetylated CRYAB, PARK7, S100A4, and ACTG1. Despite more pronounced early allograft dysfunction in DCD recipients, DCD tissue proteoform landscapes were globally more similar to LD than to DBD kidneys, indicating distinct injury biology not captured by clinical metrics or peptide-centric proteomics. To extend these findings beyond the graft, we performed the first paired tissue and PBMC proteoform profiling across donor types. Baseline PBMC proteoform differences were minimal; however, by day 7 post-transplant, recipients of deceased donor kidneys exhibited platelet activation, cytoskeletal stress, and inflammatory signaling. Several PBMC-derived proteoforms correlated with serum creatinine, highlighting their potential as early, objective indicators of graft adaptation. Collectively, this proof-of-concept study defines conserved injury-associated proteoforms across tissue and immune compartments and supports the idea that proteoform-resolved analysis may provide a mechanistically grounded, objective framework for assessing donor kidney quality and early graft function.

## Highlights

• First paired donor tissue and recipient blood proteoform analysis.
• Proteoform signatures distinguish living, deceased, and discarded donor kidneys.
• Stress-associated proteoforms are elevated in discarded and deceased donor tissues.
• Four injury-associated proteoforms are prominent across marginal donor tissues.
• Serum creatinine in recipients of poor-quality tissue correlates with kidney injury.

## Graphical Abstract

**Figure:**
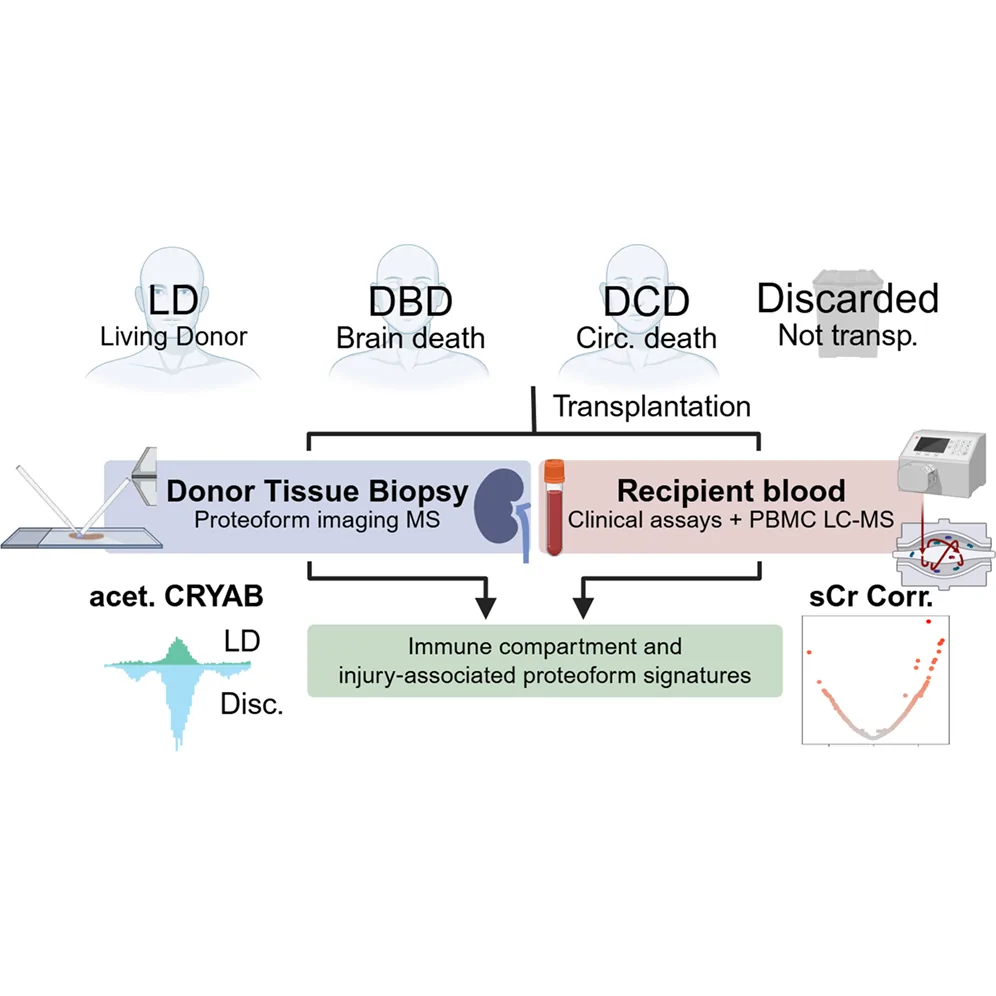

## In Brief

Donor kidney assessment lacks standardized clinical metrics, leading to high organ discard rates. We present a new proof-of-concept framework that integrates standard clinical assays with proteoform imaging mass spectrometry of donor biopsies and LC-MS-based proteoform profiling of recipient blood. This dual approach provides an objective, molecular-level evaluation of proteoforms to accurately distinguish living-donor from deceased-donor kidneys. Scaled to larger cohorts, this proteoform-resolved strategy could significantly improve donor kidney assessment quality, reduce organ discard rates, and improve long-term transplant outcomes.

Kidney transplantation (KTx) is the only available curative option for patients suffering from end-stage renal disease. Yet, there is a significant disparity between the availability of donor kidneys and the number of patients requiring KTx. According to the United States Organ Procurement and Transplantation Network, as of late 2025, more than 94,000 people are on the waitlist for a KTx (https://www.hrsa.gov/optn/data-calculators). Since the onset of KTx in 1954, traditional metrics for donor kidney evaluation have relied on medical history and demographics, histopathology of biopsies, clinical parameters such as donor urine output, serum creatinine (sCr), and estimated glomerular filtration rate (eGFR), machine perfusion parameters (*i.e*., resistive indices and flow rates), and gross inspection of the renal allograft (1, 2). While these parameters affect whether kidneys are discarded or used for transplantation, no standardized clinical assessment is currently available, and many criteria are institution- and physician-dependent. This is particularly true for donors after brain death (DBD) and donors after cardiac death (DCD), where ∼28% of the procured kidneys are discarded based on varying degrees of interstitial fibrosis and tubular atrophy, vascular disease, or diabetic changes, to name a few (3). Additionally, current allocation policies often result in long cold ischemic times for deceased donor kidneys, increasing the risk of slow and delayed graft function post-KTx and exacerbating ischemia-reperfusion injury, which poses a significant barrier to renal allograft longevity (4). These limitations underscore an unmet need to develop robust transplant-relevant point-of-care (POC) molecular markers that accurately assess donor kidney quality to improve tissue utilization and mitigate post-KTx complications.

Molecular “omics” technologies have leveraged transcriptomics, proteomics, and metabolomics to objectively assess kidney quality by complementing histopathology with donor-specific molecular signatures. However, no predictive biomarker for donor kidney quality has been successfully identified. To this end, we hypothesized that top-down proteomics (TDP) may provide reliable molecular signatures undetected by “standard” proteomics technologies (5). TDP detects intact proteoforms, which represent molecular forms of proteins, including genetic variations in protein-coding regions, post-translational modifications (PTMs), and isoforms arising from alternative splicing or translational start sites (6). Because they are more proximal to the phenotype, identification of intact proteoforms is expected to correlate more closely with the pathophysiological state of a donor organ during procurement and preservation, thereby providing an objective and reliable assessment of the donor kidney's quality (7). To date, no study has examined pre-transplant proteomic signatures as a measure of renal allograft quality.

In this study, we implemented TDP technology to identify proteoforms and pathways associated with donor kidney quality. Specifically, we probed the proteoform landscapes of human LD, DBD, and DCD kidney biopsies using proteoform imaging mass spectrometry (PiMS) and systematically investigated their impact on recipient peripheral blood mononuclear cells (PBMCs) post-KTx. We identified proteoform signatures associated with cellular stress and immune cell activation in kidney tissue biopsies and in PBMCs from recipients transplanted with deceased-donor kidneys. In contrast, we found increased metabolic regulation in donor biopsies and blood from LD recipients. These results align with clinical knowledge of LD, DBD, and DCD kidneys and different KTx outcomes, providing a proof of concept for evaluating proteoforms to assess donor kidney quality, and establishing the technical and biological feasibility of integrated multi-platform TDP profiling. When combined with traditional metrics and future cohort expansion, these techniques could significantly improve organ utilization and post-KTx outcomes.

## Methods

### Cohort and Sample Collection Information

KTx recipients were enrolled in the study at the time of transplant and included adult males or females undergoing primary or subsequent KTx from deceased donors (DD) or LD. Patients with a history of non-renal transplants, active immunosuppression (IS) therapy, or requiring desensitization prior to KTx were excluded. The study included LD recipients and standard grade DD kidneys, defined as those with a Kidney Profile Index (KDPI) of 30% to 80%. Demographic and clinical information for donors and recipients enrolled in this study are presented in Supplemental Table S1. The study period spanned from March 10, 2023, to April 20, 2024.

Resistance (PBMC cohort: median: 0.2, Q1: 0.17, Q3: 0.25; PiMS cohort: median: 0.17, Q1: 0.17, Q3: 0.25), flow rates (mL/minute; PBMC cohort: median: 135, Q1: 101.5, Q3: 144.5; PiMS cohort: median: 86, Q1: 85, Q3: 140), and cold or warm ischemic times (PBMC cohort: median: 1,031, Q1: 234, Q3: 1319; PiMS cohort: median: 1,031, Q1: 236, Q3: 1319) were similar across all groups. Induction therapy was Campath or Simulect in the PiMS (78% *versus* 22%, respectively) and PBMC (53% *versus* 47%, respectively) cohorts, similar across donor types.

The study was conducted in accordance with the Declaration of Helsinki, and the research protocol was approved by the Northwestern Medicine Institutional Review Board (IRB) under the protocol number: STU00218258. All the patients in this study consented to the use of their anonymized data, and their data are summarized in Supplemental Table S1.

Data on recipient and donor characteristics, transplant information, and post-transplant outcomes were summarized as medians (interquartile ranges, IQR) and counts (proportions) for continuous and categorical variables, respectively. Comparisons between DCD, DBD, and LD groups were analyzed with the Kruskal–Wallis rank sum test for continuous variables or Fisher’s exact test for categorical variables. A *p* value of <0.05 was considered statistically significant. All analyses were performed in R 2024.04.2 using RStudio (8).

For standard clinical metabolic testing, whole blood samples were obtained through year one post-KTx at the following time points: immediately pre-KTx, day 7, day 14, day 30, day 60, day 90, month 6, month 9, and month 12 post-KTx. Metabolic testing included serum creatinine (sCr), estimated glomerular filtration rate (eGFR), and blood urea nitrogen (BUN).

### Experimental Design and Statistical Rationale

For the relative comparison of living to discarded kidneys, one tissue section from 6 different donors (3 living, 3 discarded kidneys) was analyzed *via* PiMS. For the tissue quality comparison, a PiMS cohort (n = 9 recipients; LD: n = 3; DBD: n = 3; DCD: n = 3) and a PBMC cohort (n = 15 recipients; LD: n = 5; DBD: n = 5; DCD: n = 5), with partial overlap in donor samples (*i.e*., 8 of 9 PiMS tissues overlap with the PBMC cohort except 1 DBD donor), were acquired via PiMS.

PBMC data derived from randomized biological and technical replicates were used to assess analytical performance and differential proteoform expression across the patient groups. Missing values were filtered to retain proteoforms quantified in at least 50% of samples across at least one biological condition (*i.e*., group). PiMS data were normalized according to the respective total ion count (TIC). To stabilize variance of all TDP datasets and make them more comparable across samples, intensities were log_2_-transformed and normalized by subtracting the median intensity of each sample from all its intensities, then dividing the result by the sample standard deviation. To assess differentially expressed proteoforms, the log_2_-transformed intensities were z-score normalized across samples and used as a dependent variable in a hierarchical mixed linear regression model created using the ‘lme4’ (version 1.1.38) R package (9). Biological and technical replicates were set as random nested variants in the model, and *p*-values were adjusted using the Benjamini and Hochberg approach to control the FDR induced by multiple testing (10). In all cases, adjusted *p* values <0.05 were considered significant.

Data on recipient and donor characteristics, transplant information, and post-transplant outcomes were summarized as medians (interquartile ranges, IQR) and counts (proportions) for continuous and categorical variables, respectively. Comparisons between DCD, DBD, and LD groups were analyzed with the Kruskal–Wallis rank sum test for continuous variables or Fisher’s exact test for categorical variables. A *p*-value of <0.05 was considered statistically significant. All analyses were performed in R 2024.04.2 using RStudio (8). Pearson correlations were computed between individual proteoform intensities at Day 7 and sCr at Day 7 across patients. Only proteoforms with valid measurements in ≥3 patients were included. *p*-values were adjusted for multiple testing using the Benjamini-Hochberg procedure. Proteoforms with FDR <0.05 were considered significant.

For PiMS data, fold-changes were calculated as described above, and all regulated proteoforms were aggregated to their precursor protein and subjected to pathway analysis using Reactome (https://reactome.org/). Unsupervised hierarchical clustering of normalized proteoform abundances was performed using “ComplexHeatmap” (version 2.18.0) (11) R package. Vulcano, boxplots, bar graphs, density plots, and FDR-filtered pathways per grouped comparison were plotted using the ‘ggplot2’ (version 4.0.0) R package.

### PiMS Samples

Back-table kidney tissue biopsies were collected intraoperatively by the surgical team at Northwestern Medicine Comprehensive Transplant Center. All biopsies were obtained from isolated kidney allografts before implantation and were processed as follows: each biopsy was obtained from the upper pole of the kidney cortex to focus on inter-patient collection standardization, snap-frozen in liquid nitrogen-cooled isopentane, and stored in a cryotube at −196 °C before further processing and analysis. Additionally, three wedge biopsies from discarded kidneys (low quality and unsuitable for transplant) were included in this study. Five high-quality (living donor) adjacent cryosections (10 μm) were thaw-mounted onto a positively charged glass microscope slide (Globe Scientific) and stored at −80 °C before PiMS analysis (Optical images: Supplemental Figs. S1, S2, and S3).

Following PiMS sample pretreatment, the tissue sample was moved under the PiMS probe at a raster scanning rate of 4 μm/s with a strip step of 100 μm. PiMS utilizes nanospray desorption electrospray ionization (nano-DESI) mass spectrometry (MS) coupled with Orbitrap-based individual ion MS (I^2^MS) on an Orbitrap Exploris 480 mass spectrometer (Thermo Fisher Scientific, Bremen, Germany) (12). The nano-DESI probe is comprised of two capillaries: a primary capillary that delivers extraction solvent to the tissue, and a nanospray capillary for transfer and ionization of extracted proteoforms, interfacing with MS detection (13). Localized analyte extraction from tissue is sampled by a dynamic liquid bridge formed between the junction of the two capillaries and the tissue section. In these experiments, the tissue sample was moved under the PiMS probe at a raster scanning rate of 4 μm/s with a strip step of 100 μm, which allows proteoforms from different spatial locations to be sampled and transferred to MS for detection as individual ions (14). In all PiMS experiments, a denaturing extraction solvent (60%/39.4%/0.6% (*v*/*v/v*) acetonitrile/water/glacial acetic acid) was used at a flow rate of 400 nl/min, allowing efficient proteoform extraction and ionization in positive mode. The PiMS probe used in this study was fabricated using fused silica capillaries (Molex, Thief River Falls, MN, OD/ID 150/40 μm). To improve sampling sensitivity and spatial resolution, the primary capillary was flame-pulled to an OD of 20 μm on one side to reduce the liquid bridge diameter. A three-point plane was defined in the tissue section to compensate for the tilting angle, ensuring the stability of the liquid bridge during spatial profiling. For each tissue biopsy section, a continuous tissue area of approximately 4 mm^2^ was selected as the region of interest for PiMS spatial proteoform profiling.

PiMS experiments were conducted on the Orbitrap Exploris 480 mass spectrometer (Thermo Fisher Scientific, Bremen, Germany) in the I^2^MS mode at a resolution of 120,000 at *m/z* 200, which corresponds to 0.5 s Orbitrap detection period (a data acquisition rate of 2 spectra/s) (15). The source conditions for the mass spectrometer were set as follows: electrospray ionization voltage: 3 kV; in-source CID: 5 eV; S-Lens/Funnel RF level: 70%; capillary temperature: 325 °C. MS injection time was kept at 0.2 milliseconds to minimize “multi-ion” signals with overlapping *m/z* values from multiple individual ions in the same detection period. The injection time has been systematically optimized and benchmarked using a spare kidney tissue section from the same sample set. Other instrument parameters used in these experiments were listed as follows: Higher-energy collisional dissociation pressure setting: 0.33 (arbitrary unit); extended trapping: 0.5 V; mass range: 400 to 2500 *m/z*; AGC mode: fixed; enhanced Fourier transform: off; Emeter averaging: 0; microscans: 1.

Proteoform identification was performed using an Intact Mass Tag (IMT) approach described in detail previously (16). In brief, individual ions from the tissue were collected by the PiMS probe as a function of location; time-domain data files were acquired and recorded as Selective Temporal Overview of Resonant Ions (STORI) files (17). Proteoform features were extracted by a modified THRASH algorithm tailored for the I^2^MS datatype represented by their monoisotopic masses and ion counts (18). For proteoform quantification, we averaged the top 3 peaks of a theoretical isotopic envelope to avoid overlapping species. The relative comparison of tissue proteoforms, therefore, utilized the global ion counts across the entire acquired PiMS region rather than spatially resolved proteoform abundances. The global proteoform covariance per group and PiMS experiment was highly comparable and thus allowed for differential analysis across the tissue sections (Supplemental Fig. S4). I^2^MS mass spectrum of each biopsy was first systematically calibrated according to the accurate masses of six known abundant kidney proteoforms in the 4 to 50 kDa mass range. The calibrated spectrum was subsequently converted to .mzML format, and the proteoform features in the spectrum were searched against a custom proteoform database (259 proteoforms from healthy human kidney tissues) using TDValidator (Proteinaceous) implemented with MS (https://www.hrsa.gov/optn/data-calculators) at a ±1.5 ppm mass tolerance. Using the RStudio platform (8), TIC-normalized proteoform abundances were subjected to a nested ANOVA analysis to assess differences in proteoform expression among the three patient groups.

### PBMC Samples

Blood samples were collected at two time points, immediately pre-KTx and 7 days post-KTx, then enriched for PBMCs and subjected to liquid chromatography tandem mass spectrometry (LC-MS/MS) in technical triplicates to detect proteoforms. Pre-KTx (day 0) samples were drawn prior to the administration of any IS/induction therapies. The second sample was collected on day 7 post-KTx. CPT tubes were processed within one hour of collection and spun at room temperature at 1700*g* for 20 min.

The PBMC layer was collected and prepared as described previously (19). Briefly, the PBMC layer was collected and washed with phosphate-buffered saline, and cells were counted, diluted to a final concentration of 5 × 10^6^ cells/ml, and preserved at −196 °C in 80% fetal bovine serum/20% dimethyl sulfoxide. Cells were lysed in 20 mM Tris-HCl pH 7.5, 100 mM NaCl, 1% N-lauroylsarcoside, and protease/phosphatase inhibitor cocktails. Lysates were sonicated, proteins were precipitated, resuspended in 1% sodium dodecyl sulfate, and their concentrations were determined by BCA assay. Proteins were fractionated by Passively Eluting Proteins from polyacrylamide gels as intact species, and proteins with a molecular weight lower than 30 kDa, as described previously (20).

Protein samples were separated using a Vanquish Neo UHPLC system (Thermo Fisher Scientific) as previously described (21). Briefly, reversed-phase liquid chromatography was conducted on a MAbPac EASY-Spray column (150 mm length by 150 μm i.d., Thermo Fisher Scientific) and an in-house packed PLRP-S trap column (25 mm length by 150 μm i.d., Agilent). The total run time was 120 min, utilizing a gradient of mobile phase A (99.9% water, 0.1% FA) and mobile phase B (19.9% water, 80% acetonitrile 0.1% FA). The flow rate was maintained at 1 μl/min. The gradient profile was as follows: 5% B at 0 min, 20% B at 5 min, 70% B at 110 min, 99% B from 111 to 114 min, and 5% B from 115 to 120 min. The column outlet was connected in line to an EASY-Spray source on an Orbitrap Ascend mass spectrometer (Thermo Fisher Scientific) operating in intact protein mode with 2 mTorr of N_2_ pressure in the ion routing multipole. The transfer capillary temperature was set to 320 °C, the ion funnel RF was set to 60%, and a source collision induced dissociation (CID) of 15 V was applied. MS1 spectra were acquired at resolution of 120,000 power (at m/z 200), normalized AGC target of 500%, maximum injection time 100 ms and 1 μscan. Target precursors were isolated using a 3 m/z window for data-dependent topN MS2 method included 60,000 (at m/z 200), resolution, 27 NCE for HCD with a normalized AGC of 800%, 800 ms maximum injection time and 1 microscan with a dynamic exclusion of 60 s and a threshold of 1 × 10^4^ intensity.

The raw data files were analyzed using the publicly accessible standard workflow on TDPortal (https://portal.nrtdp.northwestern.edu, Code Set 4.0.0). This workflow conducts mass inference and searches against a database of human proteoforms, derived from SwissProt (2.4 million entries; June 2020), with curated histones, maintaining a 1% FDR at the protein, isoform, and proteoform levels (22). For quantitative proteoform analysis, a CSV intensity sheet was generated using an isotopic-fitting algorithm across the chromatogram to determine the intensities of all proteoforms identified in the study, with 1% FDR confidence in each sample.

## Results

### Study Design and Cohort Overview

To characterize the proteoform landscape of donor kidneys, using direct tissue sampling and its downstream effect on recipients through blood proteoforms and clinical metabolic markers, we enrolled patients at the time of kidney transplantation across two cohorts: a PiMS cohort (n = 9 recipients; LD: n = 3; DBD: n = 3; DCD: n = 3) and a PBMC cohort (n = 15 recipients; LD: n = 5; DBD: n = 5; DCD: n = 5), with partial overlap in donor samples (Fig. 1*A* and Supplemental Table S1; 8 of 9 PiMS tissues overlap with PBMC cohort except 1 DBD donor). Recipient median age was 53 years (IQR 50, 60) and 60 years (IQR 52, 61), respectively, and most were male (89% and 73%, respectively). The most common disease etiology was diabetes mellitus (33% and 56%, respectively). Two patients in the PBMC cohort (13%-DBD recipients) and one in the PiMS cohort (33%-DBD recipient) had previous transplants. Most patients were on dialysis pre-KTx (77.8% and 73.3%, respectively), predominantly hemodialysis *versus* peritoneal dialysis, with median dialysis duration of 2.7 years (IQR 1.8, 3.4) and 3 years (IQR 1.0, 3.7), respectively. Median donor age was 51 years (IQR 45-58, IQR 37-58), approximately half male, with characteristics balanced across donor types. Full cohort inclusion criteria, technical methodology, and IRB approval details are provided in the sample and cohort information within the methods section.

**Fig. 1:**
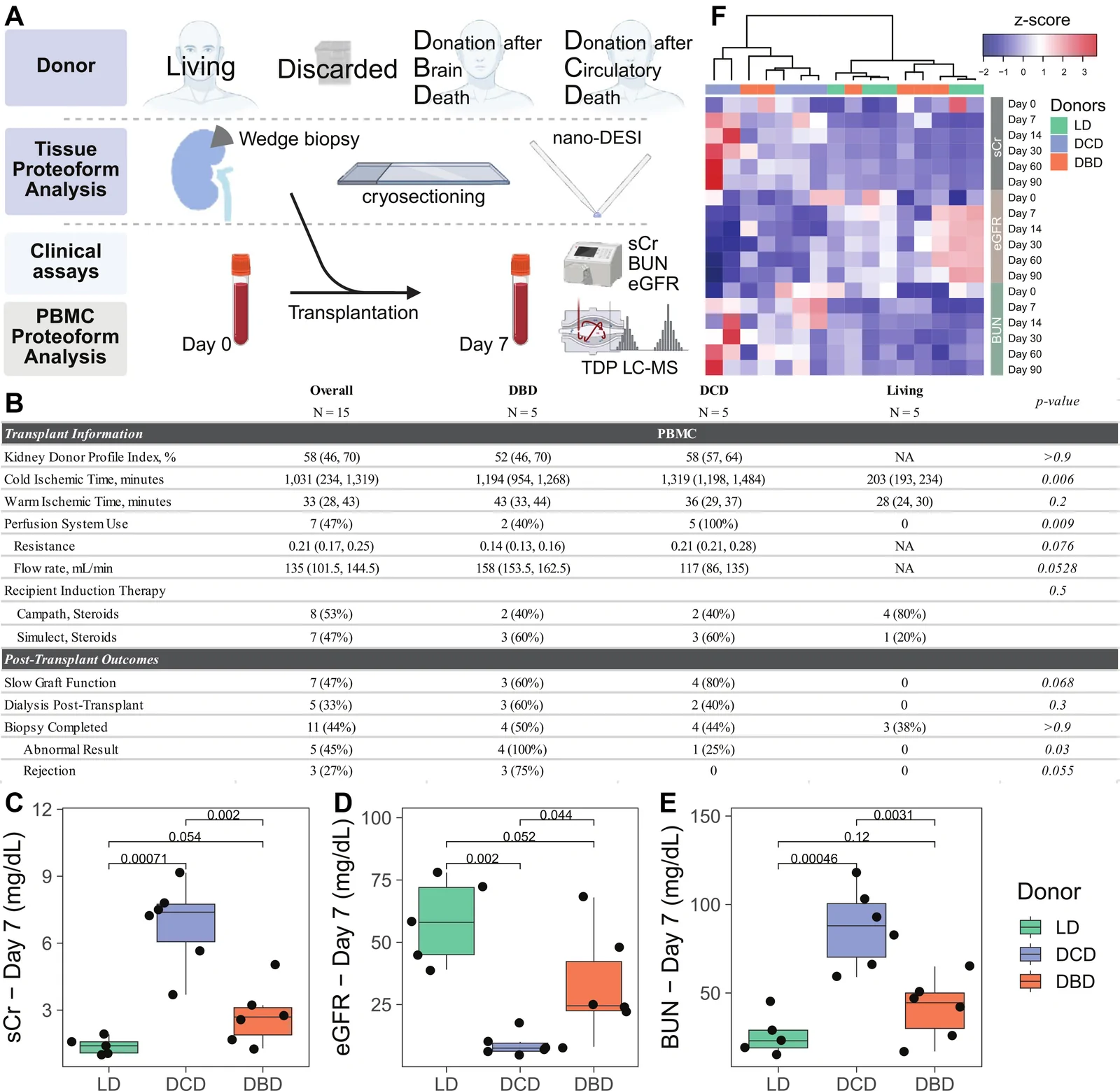
**Standard clinical assays indicate higher similarities between LD and DBD recipients.***A*, multilevel kidney proteoform profiling across living (LD), discarded (Disc.), Donation after Brain Death (DBD), and Donation after Circulatory Death (DCD) donor kidneys. nano-DESI = nanospray desorption electrospray ionization for direct tissue proteoform analysis; TDP LC-MS = *top-down* proteomics via liquid chromatography mass spectrometry for proteoform analysis. sCr = serum creatinine, BUN = blood urea nitrogen, eGFR = estimated glomerular filtration rate. Created in BioRender. https://BioRender.com/60jyi32. *B*, overview of transplant and post-transplant outcomes for all recipients. *C–E,* Relative comparison of (*C*) sCr, (*D*) eGFR, and (*E*) BUN levels at day 7 post-KTx for LD, DCD, or DBD recipients with ANOVA-based *p*-values (n = 6). *F*, hierarchical clustering of BUN, eGFR, and sCr levels for LD, DCD, or DBD recipients on days 0, 7, 14, 30, 60, or 90 post-KTx.

Deceased donors show a median KDPI of 58% (IQR 40-63, IQR 46-70) (Fig. 1*B* and Supplemental Table S1), and approximately half (100% of DCD kidneys) underwent machine perfusion pre-implantation. In the PBMC cohort, slow graft function (SGF) (23) was diagnosed in 47% of recipients, and post-KTx dialysis was required in 33%, exclusively in DD recipients, with similar early graft function outcomes in the PiMS cohort. Surveillance and for-cause biopsies within the first year revealed abnormal pathology in more than 60% of biopsied patients (PBMC 73%, PiMS 67% of all patients), including moderate arteriosclerosis, primary disease recurrence, and/or tubule-interstitial cellular infiltrates (Supplemental Table S1). Antibody-mediated or cellular rejection was observed in 27% and 17% of the PBMC and PiMS cohorts, respectively, despite standardized IS regimens at higher rates in DBD recipients.

**Fig. 2:**
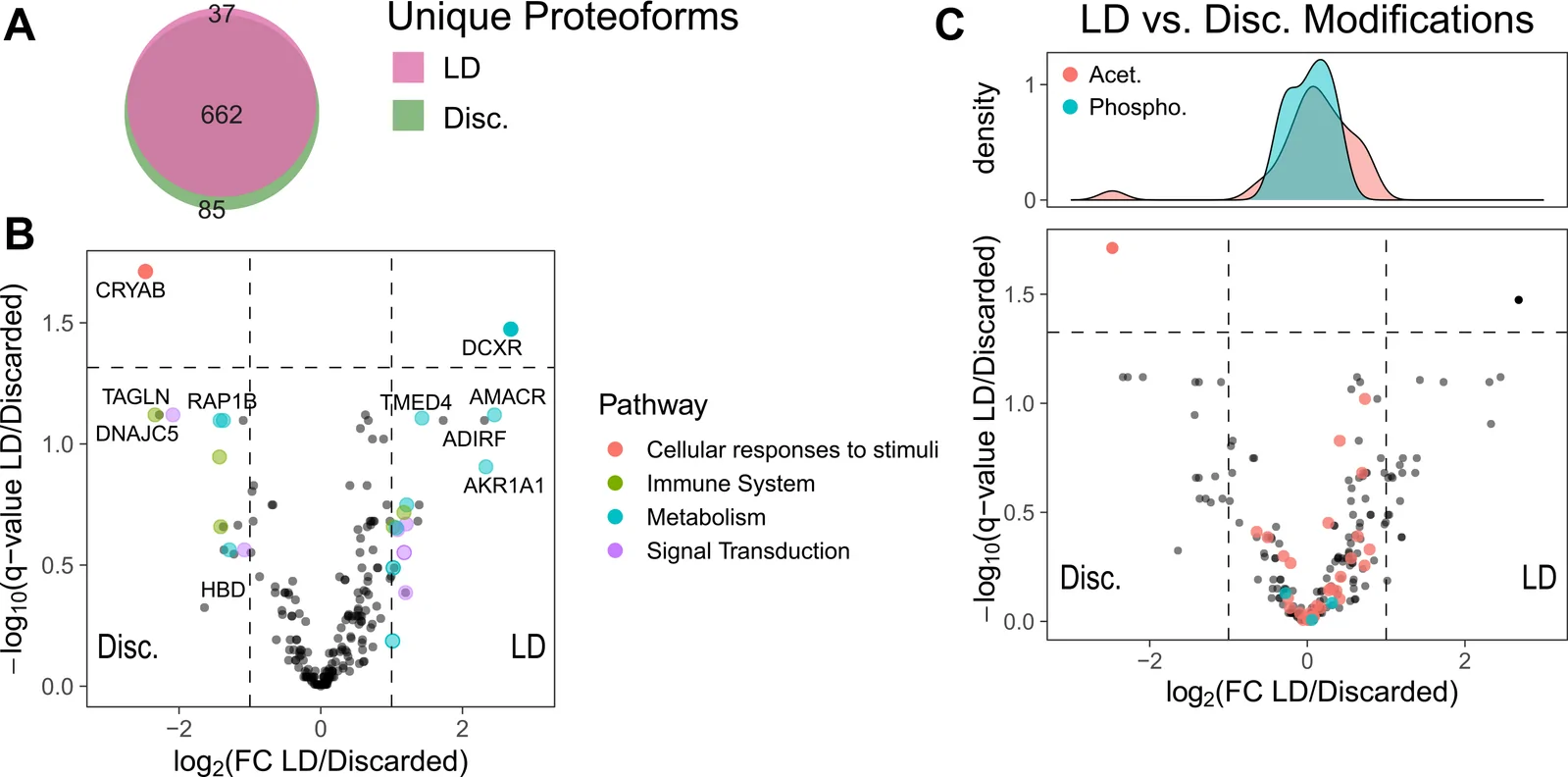
**Tissue proteoform imaging reveals elevated levels of cellular stress and immune regulation in discarded *versus* transplanted kidneys.***A*, Venn diagram of LD and discarded kidney tissue proteoforms based on PiMS analysis. *B*, Log_2_ fold-change and –log_10_ q-value volcano plot of LD *versus* discarded proteoforms; top 4 regulated pathways are color-coded and labeled with their parent proteins. *C*, acetylated (acet.) or phosphorylated (Phospho.) proteoforms are highlighted in the inset atop the volcano plot (contains the same data as in *panel B*).

**Fig. 3:**
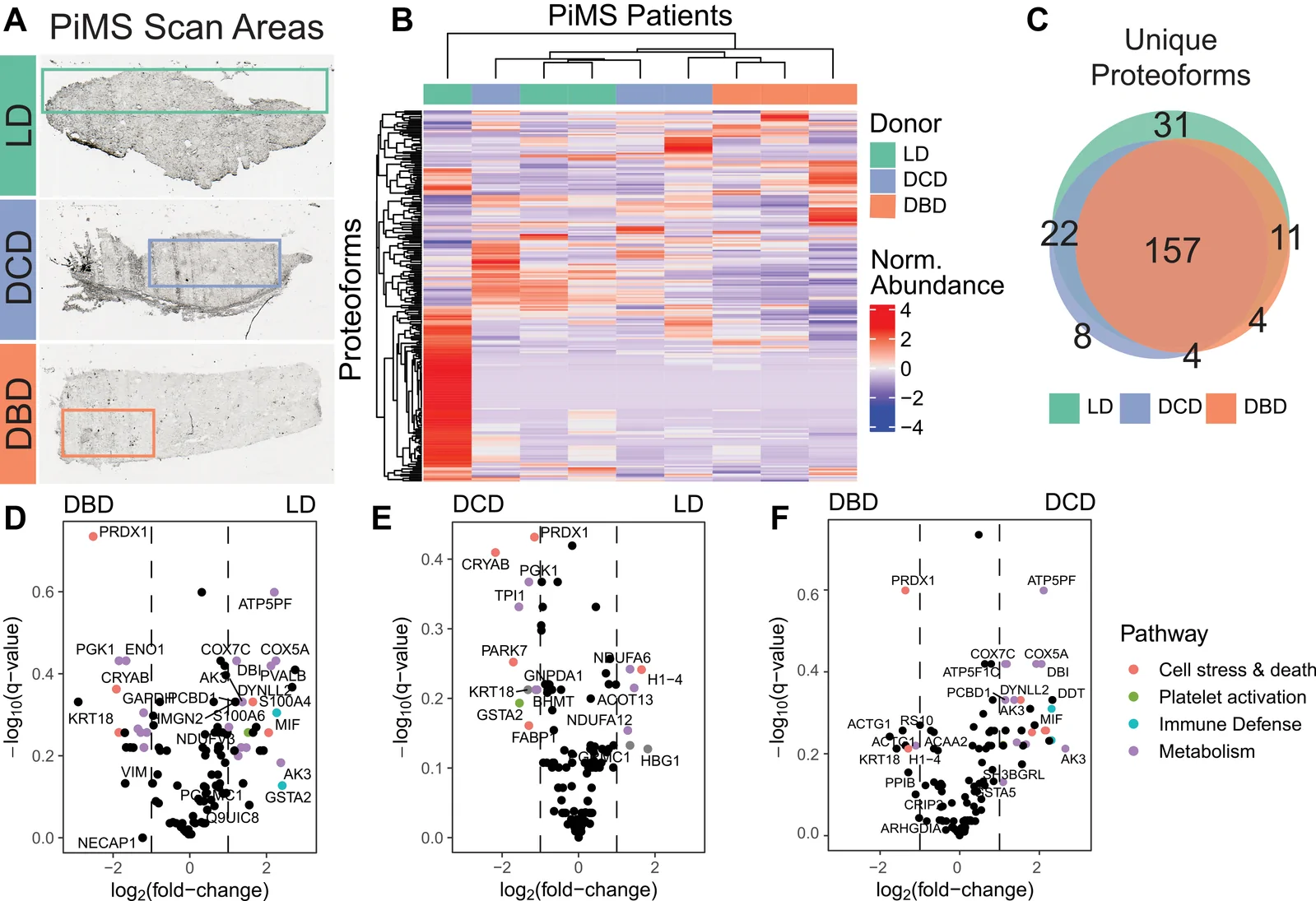
**Tissue proteoforms from DCD and DBD donors indicate higher molecular similarity between DCD and LD kidneys.***A*, representative tissue sections with highlights of the regions acquired with PiMS. *B*, hierarchical clustering of normalized proteoform abundances, colored by donor group and recipient outcome. (*C*) Venn diagram of unique proteoform overlap for LD, DCD, and DBD donors. *D–F*, Log_2_ fold-change and –log_10_ q-value volcano plots of (*D*) LD *versus* DBD, (*E*) LD *versus* DCD, and (*F*) DCD *versus* DBD. Colors indicate representative pathways, including cell stress, platelet activation, immune defense, and metabolism (Reactome pathways detailed in the Supplemental Methods). Proteoforms are labeled with their source protein.

### Donor Type Determines Early Graft Recovery Post-kidney Transplantation

To establish the clinical context for molecular profiling, we first evaluated early graft recovery across LD, DBD, and DCD recipients (Fig. 1*A*). Kidney function was monitored from pre-KTx (day 0) through 12 months post-KTx using standard metabolic parameters (*i.e*., sCR, BUN, and eGFR) across 15 patients with distinct clinical characteristics (Cold Ischemic Times, Recipient Induction Therapy and Post-transplant Dialysis Fig. 1*B*). As expected, LD recipients demonstrated rapid normalization of sCR, BUN, and eGFR within the first week post-KTx (Fig. 1, *C–E*). In contrast, DD recipients exhibited delayed return to baseline, with DCD recipients showing the highest sCR levels at day 7 post-KTx, while DBD recipients displayed intermediate metabolic recovery (Fig. 1, *D* and *E*). Early recovery kinetics were further corroborated by hierarchical clustering of metabolic parameters monitored up to day 90 post-KTx, which separated deceased donors from LD recipients and further distinguished DCD and DBD recipients based on recovery trajectory (Fig. 1*F*). Together, these findings establish that donor type is a strong determinant of early graft recovery, yet the molecular basis underlying this divergence remains uncharacterized, motivating direct proteoform-level investigation of donor kidney tissue.

### PiMS Identifies Distinct Molecular Profiles in Living and Discarded Donor Kidney Biopsies

To determine whether tissue-level proteoform composition reflects donor kidney quality, we performed PiMS on biopsies from LD obtained at procurement (high quality) and discarded kidneys unsuitable for transplantation (low quality). Comparative analysis revealed a total of 784 proteoforms, with 37 unique to LD biopsies, 85 unique to discarded biopsies, and 662 common to both (Fig. 2*A*). Pathway enrichment analysis identified twenty upregulated proteoforms that were metabolism-related in LD, while 16 significantly elevated proteoforms in discarded biopsies were associated with cellular stress and immune regulation (Fig. 2*B*). Global PTM analysis revealed an equal distribution across both groups, with alpha crystallin B-chain (CRYAB), a stress response chaperone, as the most differentially regulated acetylated proteoform in the discarded biopsies (Fig. 2*C*). Collectively, these findings indicate that intact proteoform composition distinguishes donor kidneys suitable for transplantation from those that are not, and suggest that cellular stress remodeling is a defining feature of poor-quality donor tissue.

### Tissue Proteoform Analysis Reveals Differentially Expressed Proteoforms Associated With Oxidative Stress in Deceased Donors

Informed by clinically suitable and non-transplantable donor kidney profiles, we next performed PiMS on transplant-suitable LD, DBD, and DCD biopsies to determine whether donor-type specific proteoforms could inform donor-kidney quality (Fig. 3*A*). Across 393 identified proteoforms, hierarchical clustering separated proteoforms in LD and DCD biopsies from DBD biopsies (Fig. 3*B*). We identified 157 proteoforms shared among all donor types, while LD biopsies exhibited the highest number of unique proteoforms (n = 31), compared with DCD biopsies (n = 8) and DBD biopsies (n = 4) (Fig. 3*C*). LD and DCD biopsies shared 22 proteoforms not observed in DBD biopsies, whereas only 4 proteoforms were uniquely shared between DBD and DCD biopsies. This indicates higher molecular similarity between LD and DCD than between LD and DBD biopsies.

Compared to LD biopsies, 12 and 19 proteoforms were significantly more abundant in DCD and DBD biopsies, respectively. While LD kidneys were enriched for oxidative phosphorylation components such as COX5A and COX7C, DCD biopsies were enriched for stress-associated proteoforms, including PRDX1, CRYAB, and S100A4, alongside metabolic regulators such as NDUFV3 and GAPDH (Fig. 3, *D–F*). DBD biopsies were enriched for proteoforms linked to cellular stress and cytoskeletal remodeling, including ACTG1, ATP5PF, and MIF (Fig. 3*D*). A direct comparison of DBD and DCD biopsies highlighted proteoform divergence in deceased donors. DBD biopsies demonstrated relative enrichment of proteoforms associated with cytoskeletal organization and immune signaling, whereas DCD biopsies retained a higher abundance of several metabolic proteoforms (Fig. 3*E*). Together, these data indicate that while both DD types exhibit increased cellular stress relative to LD biopsies, DBD biopsies display more pronounced cytoskeletal and immune-associated remodeling, whereas DCD biopsies preserve metabolic proteoform signatures overlapping with LD biopsies (Fig. 3, *D–F*). These findings suggest that donor-specific proteoform signatures at the time of transplantation could inform differences in early graft recovery.

### Integration Across Discarded and Deceased Donor Cohorts Identifies Conserved Injury Proteoforms

To identify proteoforms most consistently associated with impaired donor kidney quality, we integrated the molecular profiles of all donors. We found substantial proteoform intersections across all donor types (178), with a discrete subset of proteoforms shared across discarded and DD biopsies relative to LD biopsies (Fig. 4*A*). The four shared proteoforms were identified as acetylated CRYAB, acetylated PARK7, acetylated S100A4, and ACTG1 (Fig. 4, *B* and *C*). Notably, three of these, CRYAB, PARK7, and S100A4, were differentially regulated between LD and both deceased and discarded donor tissues, consistent with their established roles in stress response, oxidative sensing, and immune cell recruitment, respectively. ACTG1, a marker of actin cytoskeletal reorganization and cellular activation, was predominantly elevated in DBD kidneys, even relative to DCD, suggesting engagement of cytoskeletal pathways (24). The distinct activation of these pathways in both discarded and transplant-suitable deceased donor biopsies suggests a continuum of tissue injury severity rather than a binary discard-*versus*-use distinction (Fig. 4*C*). These findings define conserved injury-associated proteoforms detectable at procurement, which distinguish LD biopsies from both discarded and deceased-donor biopsies and supports their potential for organ prioritization in future validation studies aimed at objective assessment of donor kidney quality.

**Fig. 4:**
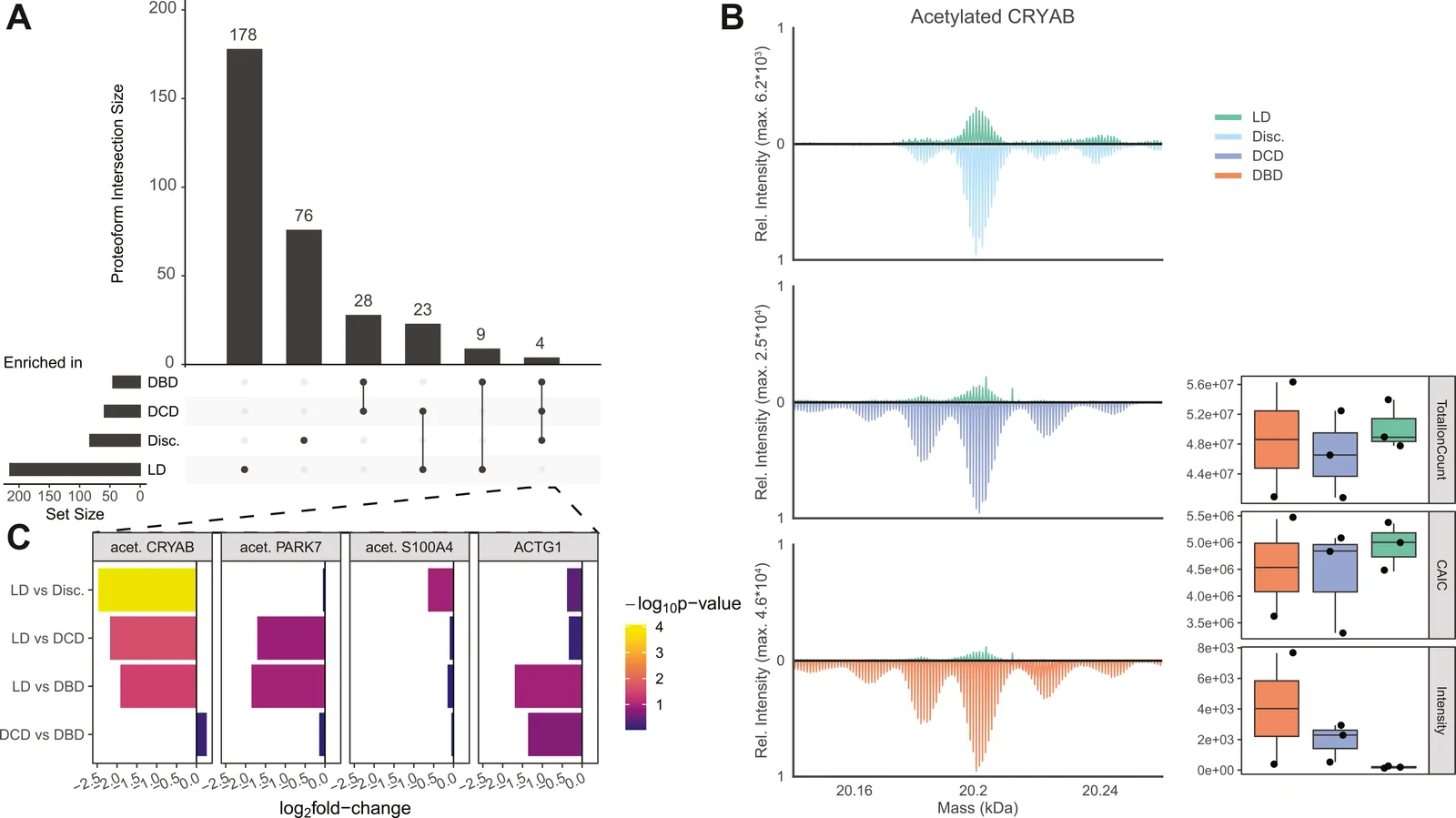
**Proteoform overlaps highlight possible markers for tissue quality.***A*, upset plot of abundant proteoforms as observed in DBD (*versus* LD), DCD (*versus* LD), discarded, Disc. (*versus* LD), or LD (*versus* DBD, DCD, and disc.). *B*, mass spectra of acetylated CRYAB (20,188 Da) for LD *versus* Discarded, DCD, or DBD. Relative intensity with the respective maximum intensity in the indicated comparison and the mass range (kDa) is shown. The boxplots indicate the quantification across the individual bioreplicates, including the TIC, CAIC (charge assigned ion count), and the intensity. *C*, Log_2_ fold-change of 4 shared proteoforms (standard error: acet. CRYAB: LD *versus* DCD/LD *versus* DBD/DCD *versus* DBD: 0.549; acet. PARK7: LD *versus* DCD/LD *versus* DBD/DCD *versus* DBD: 0.954; acet. S100A4: LD *versus* DCD/LD *versus* DBD/DCD *versus* DBD: 0.859; ACTG1: LD *versus* DCD/LD *versus* DBD/DCD *versus* DBD: 0.866) between DD and the discarded kidney across LD *versus*. Discarded, DCD, or DBD, and DCD *versus*. DBD. Color indicates -log_10_ adjusted *p*-value.

### Donor Kidneys are Associated with Distinct Early PBMC Molecular Landscapes in Recipients

To determine whether donor kidney quality affects the molecular landscape in recipients post-transplantation, we analyzed PBMC proteoform profiles at day 0 (pre-KTx) and day 7 post-KTx using TDP LC-MS/MS. Per timepoint, PBMC proteoform abundance did not segregate by donor type (Fig. 5*A* and Supplemental Fig. S6), and the large 34.9% overlap across the groups indicates comparable pre-KTx proteoform landscapes (total proteoforms 3570 in LD, 5926 in DCD, and 5702 in DBD identified; Fig. 5*B*). Strikingly, proteoform abundance changed by day 7 and clearly separated deceased donor recipients from LD recipients (Fig. 5*C*). This is supported by the large overlap of uniquely identified proteoforms between the deceased donor recipients (30%), *versus* only 3.4% (DBD) or 3.7% (DCD) compared to LD (Fig. 5*B*). Differential expression analysis confirmed the scale of this response, with substantially greater number of significantly regulated proteoforms in deceased donor recipients compared with LD recipients (LD: 2206 down-, 660 up-; DCD: 3367 down-, 2140 up-; DBD: 3548 down- and 1739 up-regulated; Fig. 5*D*). Global PTM distribution revealed that the phosphorylation patterns remained similar across recipient groups, with a slight decrease in overall acetylation 7 days post-KTx, deceased donor recipients (smaller effect in DCD than DBD; Supplemental Fig. S7), suggesting that donor-type specific changes in PBMC proteoform post-KTx did not broadly affect protein modification.

**Fig. 5:**
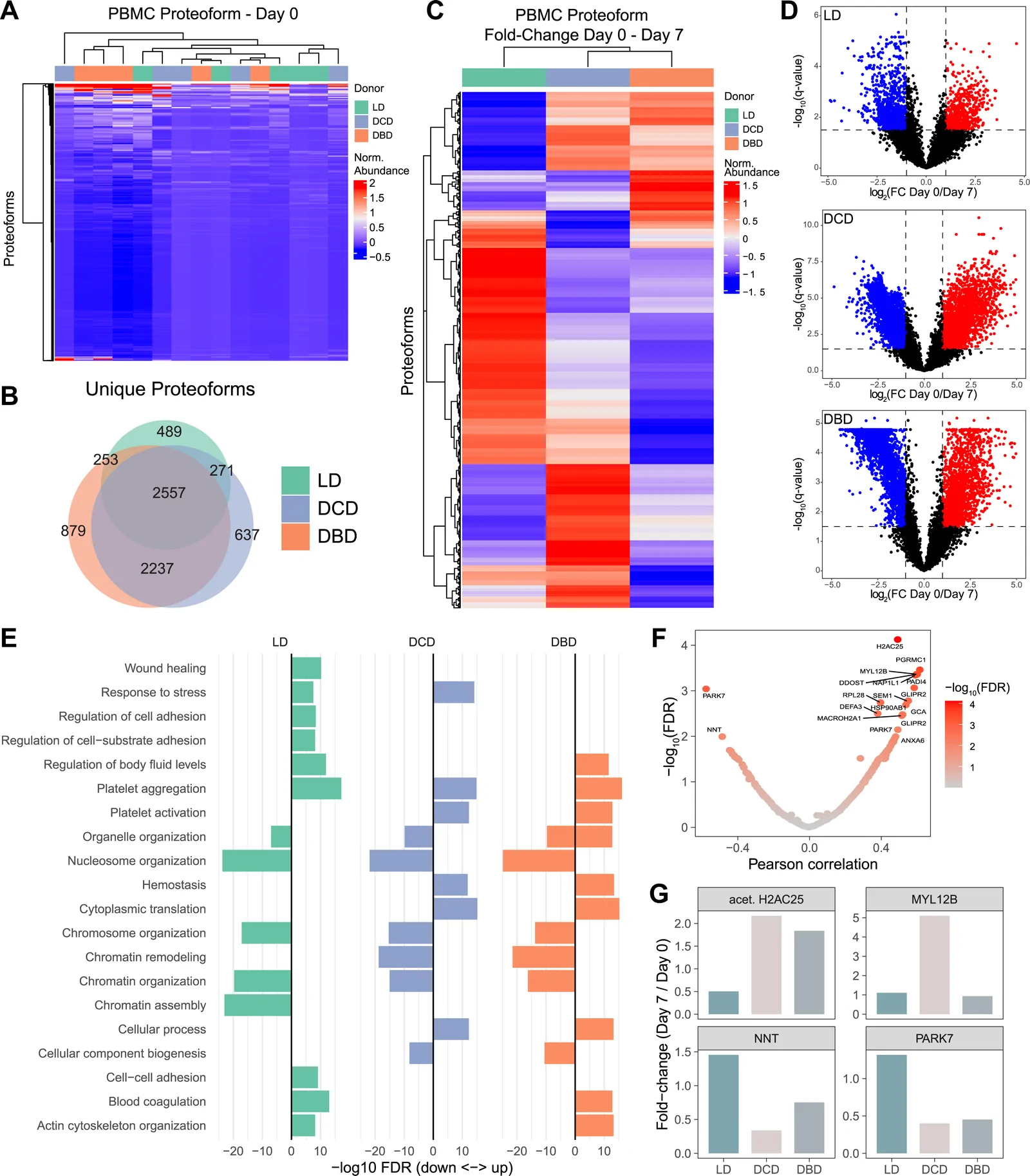
**The PBMC proteoform landscape changes drastically at 7 days post-KTx.***A*, hierarchical clustering of PBMC proteoforms prior to KTx via LC-MS analysis for all enrolled patients. *B*, Venn diagram with unique proteoform overlap. *C*, fold change of day 0 *versus* day 7 recipients with the respective patients’ clinical outcome. *D,* differential proteoform expression pre- and 7 days post-KTx for LD, DCD, and DBD. *E*, pathway enrichment analysis of significantly regulated proteoforms per recipient group (LD, DCD, and DBD). *F*, correlation of the blood sCr level and PBMC proteoforms on day 7. Source proteins are labeled. *G*, proteoform fold-changes pre- and 7 days post-KTx of the top 2 most significantly correlating (acet. H2AC25 and MYL12B) or anti-correlating (NNT and PARK7) proteoform abundances relative to day 7 sCr.

Pathway enrichment analysis of significantly regulated proteoforms further highlighted donor kidney quality changes in PBMC proteoforms (Fig. 5*E*). While deceased donor recipients demonstrated enrichment for pathways related to platelet activation, blood coagulation, cytoplasmic translation, and response to stress, LD recipients highlighted wound healing, cell adhesion, and actin cytoskeleton organization. Downregulated pathways across groups were particularly prominent in chromatin assembly and nucleosome organization, suggesting coordinated epigenetic and structural remodeling during early post-transplant adaptation (Fig. 5*E*). Indeed, a 2D-pathway enrichment analysis combining proteoforms from DD recipients revealed cell stress and immune defense proteoforms compared to metabolism-associated proteoforms in DCD and LD donors (Supplemental Fig. S8, *A* and *B*). Together, these findings demonstrate that donor kidney quality impacts the early recipient PBMC molecular landscape post-KTx, with distinct proteoform landscapes and biological pathways in DD recipients.

### PBMC Proteoforms Correlate with Early Graft Dysfunction

To assess the clinical relevance of early PBMC proteoform remodeling, we correlated day 7 proteoform abundances with contemporaneous sCr levels in LD and DD recipients. Pearson correlation analysis identified multiple proteoforms significantly associated with sCr. These sCr-associated proteoforms were predominantly observed in DD recipients, consistent with the greater abundance of proteoforms in this group (Fig. 5*F*). Among these, acetylated H2AC25, a core nucleosome component, and MYL12B, previously associated with septic acute kidney injury (25), demonstrated positive correlations with sCr in DD recipients (Fig. 5*G*). Conversely, NNT and PARK7, both linked to oxidative stress, exhibited inverse correlations with sCr in LD recipients. This indicates that donor kidney quality may distinctly influence early PBMC proteoform abundance and that specific candidates correlate with early graft function.

## Discussion

Reliable molecular markers to guide donor kidney assessment and reduce the ∼30% discard rate remain an unmet clinical need. Current evaluation methods remain subjective and fail to capture the multifactorial processes of cellular injury that determine post-KTx outcome. To address this, using a novel TDP approach, we generated the first paired proteoform insights of donor kidney biopsies and recipient PBMCs to identify proteoform signatures associated with donor kidney quality and early post-transplant adaptation.

LD kidneys represent the gold standard for organ quality. PiMS analysis demonstrated that LD kidneys are enriched in metabolically active proteoforms and relatively depleted in stress-associated proteoforms, consistent with their minimal ischemic and metabolic burden and favorable clinical outcomes (26). In contrast, both DBD and DCD kidneys showed enrichment of stress-responsive proteoforms, including acetylated CRYAB and S100A4, which were also elevated in discarded organs. These proteoforms therefore represent candidate indicators of tissue stress that are independent of donor classification and may reflect shared injury pathways across DD types. Surprisingly, we found greater proteoform similarity between LD and DCD kidneys than between LD and DBD biopsies. Differential expression and pathway enrichment analyses, however, revealed differences between LD and DCD kidneys, indicating that proteoform similarity does not imply molecular equivalence. The proteoform similarity likely reflects mechanistic differences in injury mechanisms underlying each donor type. DCD kidneys sustain acute warm ischemic injury, leaving a metabolic signature more analogous to LD tissue, whereas DBD kidneys are exposed to brain death-associated systemic inflammation and endothelial activation that drive widespread remodeling of the tissue proteoform landscape (13, 14, 15, 20, 27). These molecular distinctions are not fully captured by the current clinical metrics and may partly explain why DCD kidney outcomes are more variable than their KDPI scores suggest.

Beyond detecting local allograft changes, our paired PBMC proteoform analysis demonstrates that donor kidney quality is associated with distinct early PBMC proteoform landscapes in recipients. Baseline profiles did not segregate by donor type, supporting comparable status across groups. By day 7, however, PBMC profiles diverged sharply by donor type. DD recipients exhibited broad upregulation of homeostasis and platelet activation pathways, highlighting the complex interplay between graft quality and systemic immune adaptation. In contrast, LD recipients demonstrated enrichment of wound healing and cellular repair pathways, reflecting fundamentally different adaptive responses. Notably, global PTM distributions remained stable despite these differences, indicating that early systemic adaptation reflects selective proteoform reprogramming rather than widespread dysregulation. These insights establish that donor kidney quality leaves a detectable molecular imprint on recipient's blood proteomes during the critical first week post-KTx, potentially enabling early detection of graft dysfunction, guiding IS strategies, and improving long-term outcomes. Importantly, several proteoforms linked to oxidative stress sensing and redox homeostasis were identified in DD and recipient PBMCs, suggesting that redox remodeling represents a shared axis of donor injury and systemic adaptation. This convergence supports a model in which ischemic and inflammatory stress states are encoded at the proteoform level and propagated systemically following transplantation. Furthermore, correlation of specific PBMC proteoforms with sCr at day 7 supports the biological relevance of donor kidney quality post-KTx. While exploratory, these associations suggest that early systemic proteoform signatures may reflect biologically meaningful graft-host interactions.

While proteoform analysis has successfully evaluated donor kidney quality and its impact on KTx (28, 29), we demonstrated here that TDP has several key advantages. Unlike bottom-up proteomics, TDP analysis captures these markers in their biologically active, post-translationally modified forms, providing mechanistic specificity that peptide-level approaches cannot. Additionally, while TDP requires specialized MS infrastructure for discovery, identified candidates are readily translatable into immunohistochemistry panels deployable during routine organ procurement and preservation assessment. Thus, proteoform-resolved profiling using TDP provides a biologically informative framework for identifying molecular features of donor kidney quality and early post-transplant adaptation with clinical applicability.

This study is limited by its exploratory design and modest cohort size, reflecting a proof-of-concept demonstration rather than a validated clinical study. While cold/warm ischemic time, perfusion parameters, and induction immunosuppression regimen did not differ significantly across donor groups, recipient-level heterogeneity, including re-transplant status and individual IS responses may still contribute to PBMC proteoform variability and cannot be fully differentiated at this sample size. Longitudinal profiling of recipient PBMC proteoforms beyond day 7 was not performed, and mechanistic validation of individual proteoforms was outside the scope of this work. Additionally, tissue biopsies were consistently sampled from the upper pole cortex to standardize collection across donors, which inherently does not capture glomerular-specific biology and may not fully represent whole-organ quality. Nevertheless, the consistent findings across donor kidney biopsies and recipient PBMCs, and the alignment of proteoform signatures with established clinical and pathological outcomes, support the biological robustness of these results.

A key limitation of current TDP workflows is the complexity of proteoform annotation through combinatorial modifications and sequence diversity. The generation of a sample- or donor-group-specific bottom-up LC-MS/MS database could reduce the search space to high-confidence proteoform identifications that were identified with complementary strategies. Moreover, the integration of bottom-up and top-down data at the pathway level could leverage the depth of bottom-up coverage alongside the modification- and isoform-level resolution unique to the TDP datasets. This hybrid bottom-up/top-down strategy could represent a practical path toward maximizing biological coverage while preserving the proteoform-level specificity in future studies with expanded cohort sizes.

In conclusion, TDP reveals distinct proteoform abundance and remodeling in different transplantable donor kidneys and demonstrates that these changes are associated with early systemic adaptation in transplant recipients. The cross-cohort tissue candidates and sCr-associated PBMC proteoforms identified here represent a prioritized set for clinical validation as objective markers of donor kidney quality and early predictors of transplant outcomes. Future validation in larger cohorts will establish whether proteoform-guided donor organ quality assessment can reduce discard rates, stratify IS strategies, and ultimately improve long-term transplant outcomes.

## Data Availability

The mass spectrometry datasets generated for this study were deposited in MASSIVE (https://massive.ucsd.edu/ProteoSAFe/) with the identifier: MSV000100691.

## Supplemental Data

This article contains supplemental data.

## Conflict of Interest

The authors declare the following financial interests/personal relationships which may be considered as potential competing interests: S. N. N. serves as the Chief Medical Advisor for Pandorum Technologies Pvt Ltd and on the Scientific Advisory Board for TransMedics Inc. N. L. K. reports a conflict of interest with I2MS technology, being commercialized by Thermo Fisher Scientific. N. L. K. is involved in the commercialization of data analysis software and is a paid consultant for Thermo Fisher Scientific.
