# Supplementary material for: Top-Down Proteomics in the Assessment of Kidney Donor Quality: A Proof of Concept for Increased Organ Utilization
Source: Mol Cell Proteomics. 2026 Sep 19;25(10):101664. doi: 10.1016/j.mcpro.2026.101664 (PMC13692162; doi:10.1016/j.mcpro.2026.101664)
Supplement: Supplementary file 1 [file mmc7.docx]

Supplemental Data to

**Top-Down Proteomics in the Assessment of Kidney Donor Quality: a novel approach to increased organ utilization**

Claudia Ctortecka^1#^, Dinesh Jaishankar^2,3#^, Pei Su^4,5,6^, Che-Fan Huang^4^, Indira Pla^4^, Nathaniel Henning^4^, Michael A. R. Hollas^4^, Michelle A. Callegari^2,3^, Meredith E. Taylor^2,3^, Yu Min Lee^2,3^, Amna Daud^2,3^, David F. Pinelli^2,3^, Vinayak Rohan^2,3^, Michael A. Caldwell^1,4^, Eleonora Forte^7^, Aniel Sanchez^4,5^, Neil L. Kelleher^1,4,5,8*^  and Satish N. Nadig^2,3,9*^.

#Equal contribution

^*^Co-corresponding authors

**Affiliations**

1. Chemistry of Life Processes Institute, Northwestern University, Evanston, IL
2. Department of Surgery, Division of Organ Transplant, Feinberg School of Medicine, Northwestern University, Chicago, IL
3. Comprehensive Transplant Center, Feinberg School of Medicine, Northwestern University, Chicago, IL
4. Proteomics Center of Excellence, Northwestern University, Evanston, IL
5. Department of Chemistry, Northwestern University, Evanston, IL
6. Department of Biochemistry, University of California, Riverside, CA
7. Division of Nephrology, Department of Medicine, College of Medicine, University of Illinois Chicago, Chicago, IL
8. Department of Molecular Biosciences, Northwestern University, Evanston, IL
9. Department of Microbiology-Immunology and Pediatrics, Feinberg School of Medicine, Northwestern University, Chicago, IL

**Correspondence**

Drs. Satish N. Nadig and Neil L. Kelleher

[satish.nadig@nm.org](mailto:satish.nadig@nm.org), [n-kelleher@northwestern.edu](mailto:n-kelleher@northwestern.edu)

# **Supplemental Figures**

**Supplementary Figure S1.** Optical images of the exact areas selected and the extracted tissue areas for each biopsy in the LD cohort.

**Supplementary Figure S2.** Optical images of the exact areas selected and the extracted tissue areas for each biopsy in the DBD donor cohort.

**Supplementary Figure S3.** Optical images of the exact areas selected and the extracted tissue areas for each biopsy in the DCD donor cohort.

**Supplementary Figure S4.** Log_2_ proteoform quantities per patient are shown without normalization (unnormalized), normalized by TIC, the acquired tissue area (Tissue Area Normalized), THRASH TIC, the most abundant THRASH features, or total ribosome proteoform signal.

**Supplementary Figure S5.** Post-KTx outcomes for all patients included in the PiMS experiments.

**Supplementary Figure S6:** Hierarchical clustering of PBMC proteoforms 7 days post-KTx via LC-MS analysis for all enrolled patients.

**Supplementary Figure S7.** Log_2_ fold-change of LD, DBD, and DCD for pre- and day 7 post- KTx and –log_10_ q-value indicating acetylation and phosphorylation patterns.

**Supplementary Figure S8. (a)** 2D enrichment of PBMC proteoform fold-changes (day 0 to day 7) LD relative to DCD (x-axis) or DBD (y-axis). Selected pathways are indicated for cell stress and death, platelet activation, immune defense, and metabolism (summarized pathways are detailed in supplementary methods). **(b)** Fold change of selected immune response-associated proteoforms for LD, DCD, and DBD recipients; border color indicates patient outcome. **(c)** Pearson correlation of proteoform abundances with sCr at day 7 across LD, DCD, and DBD recipients. Colors according to -log_10_ adjusted p-value and triangles indicate histones. **(d)** Fold-changes of proteoforms with q-value <0.05 in DBD, DCD, and not LD.

**Supplementary Figure S9.** Log_2_ fold-changes across DCD versus DBD, LD versus DBD, and LD versus DCD proteoforms exclusively identified in DCD and DBD, DCD and LD, and DBD and LD biopsies. Colors indicate -log_10_ p-value.

**Supplemental Table 1**. Clinical cohort overview of PBMC and PiMS cohorts.

**Supplemental Table 2.** Proteoform quantitative table LD versus Discarded kidney tissue.

**Supplemental Table 3.** Proteoform quantitative table LD versus DCD or DBD kidney tissue.

**Supplemental Table 4.** Proteoform quantitative table of recipient blood pre- and post-transplantation of LD kidney.

**Supplemental Table 5.** Proteoform quantitative table of recipient blood pre- and post-transplantation of DCD kidney.

**Supplemental Table 6.** Proteoform quantitative table of recipient blood pre- and post-transplantation of DBD kidney.

**Supplemental Methods.**

For ease of display, the Reactome pathways displayed throughout the manuscript were grouped to ‘*Cell stress & death*’: Cellular response to chemical stress, Cellular responses to stimuli, Cellular responses to stress, Chaperone Mediated Autophagy, Late endosomal microautophagy, Selective autophagy, Defective Intrinsic Pathway for Apoptosis, Apoptosis, Regulation of Apoptosis, Apoptosis induced DNA fragmentation, Macroautophagy, Autophagy, Regulation of MITF-M-dependent genes involved in lysosome biogenesis and autophagy, FOXO-mediated transcription of oxidative stress, metabolic and neuronal genes, Cellular response to heat stress, Regulation of MITF-M-dependent genes involved in apoptosis, Turbulent (oscillatory, disturbed) flow shear stress activates signaling by PIEZO1 and integrins in endothelial cells, Intrinsic Pathway for Apoptosis, High laminar flow shear stress activates signaling by PIEZO1 and PECAM1:CDH5:KDR in endothelial cells, Response of endothelial cells to shear stress, Cellular responses to mechanical stimuli; *Metabolism*: Aerobic respiration and respiratory electron transport, Metabolism, TP53 Regulates Metabolic Genes, Glucose metabolism, Manipulation of host energy metabolism, Metabolism of carbohydrates and carbohydrate derivatives, Glycogen metabolism, Pyruvate metabolism, Regulation of pyruvate metabolism, Diseases of carbohydrate metabolism, Selenoamino acid metabolism, Metabolism of proteins, Metabolism of porphyrins, Biotin transport and metabolism, Phenylalanine metabolism, Metabolism of amino acids and derivatives, Vitamin B5 (pantothenate) metabolism, Phenylalanine and tyrosine metabolism, Metabolism of nitric oxide: NOS3 activation and regulation, Diseases of metabolism, Glyoxylate metabolism and glycine degradation, Metabolism of RNA, Triglyceride metabolism, Regulation of lipid metabolism by PPARalpha, Fatty acid metabolism, Metabolic disorders of biological oxidation enzymes, Sulfur amino acid metabolism, PI Metabolism, Metabolism of nucleotides, Arachidonate metabolism, Metabolism of water-soluble vitamins and cofactors, Phospholipid metabolism, Metabolism of vitamins and cofactors, Metabolism of lipids; *Immune Defense*: Gene and protein expression by JAK-STAT signaling after Interleukin-12 stimulation, Antigen processing-Cross presentation, Innate Immune System, MHC class II antigen presentation, Antigen processing: Ubiquitination & Proteasome degradation, Class I MHC mediated antigen processing & presentation, Adaptive Immune System; *Platelet activation*: Platelet degranulation, Platelet activation, signaling and aggregation.
